# Icariin Ameliorate Thiram-Induced Tibial Dyschondroplasia via Regulation of WNT4 and VEGF Expression in Broiler Chickens

**DOI:** 10.3389/fphar.2018.00123

**Published:** 2018-02-23

**Authors:** Hui Zhang, Khalid Mehmood, Kun Li, Mujeeb U. Rehman, Xiong Jiang, Shucheng Huang, Lei Wang, Lihong Zhang, Xiaole Tong, Fazul Nabi, Wangyuan Yao, Muhammad K. Iqbal, Muhammad Shahzad, Jiakui Li

**Affiliations:** ^1^College of Veterinary Medicine, Huazhong Agricultural University, Wuhan, China; ^2^University College of Veterinary and Animal Sciences, The Islamia University of Bahawalpur, Bahawalpur, Pakistan; ^3^College of Animals Husbandry and Veterinary Medicine, Tibet Agricultural and Animal Husbandry University, Linzhi, China

**Keywords:** tibial dyschondroplasia, icariin, thiram, WNT4, VEGF

## Abstract

Tibial dyschondroplasia (TD) is main bone problem in fast growing poultry birds that effect proximal growth plate (GP) of tibia bone. TD is broadly defined as non-vascularized and non-mineralized, and enlarged GP with tibia bone deformation and lameness. Icariin (*Epimedium sagittatum*) is a traditional Chinese medicine, which is commonly practiced in the treatment of various bone diseases. Recently, many researcher reports about the beneficial effects of icariin in relation to various types of bone conditions but no report is available about promoting effect of icariin against TD. Therefore, current study was conducted to explore the ameliorating effect of icariin in thiram-induced TD chickens. A total of 180 broiler chicks were equally distributed in three groups; control, TD induced by thiram (50 mg/kg), and icariin group (treated with icariin @10 mg/kg). All groups were administered with normal standard diet *ad libitum* regularly until the end of experiment. The wingless-type member 4 (WNT4) and vascular endothelial growth factor (VEGF) genes and proteins expression were analyzed by quantitative real-time polymerase chain reaction and western blot analysis respectively. Tibial bone parameters, physiological changes in serum, antioxidant enzymes, and chicken growth performance were determined to assess advantage and protective effect of the medicine in broiler chicken. The expression of WNT4 was decreased while VEGF increased significantly (*P* < 0.05) in TD affected chicks. TD enhanced the GP, lameness, and irregular chondrocytes, while reduced the liver function, antioxidant enzymes in liver, and performance of chickens. Icariin treatment up-regulated WNT4 and down-regulated VEGF gene and protein expressions significantly (*P* < 0.05), restored the GP width, increased growth performance, corrected liver functions and antioxidant enzymes levels in liver, and mitigated the lameness in broiler chickens. In conclusion, icariin administration recovered GP size, normalized performance and prevented lameness significantly. Therefore, icariin treatments are encouraged to reduce the incidence of TD in broiler chickens.

## Introduction

Tibial dyschondroplasia (TD) is commonly occurring leg problem in fast growing birds that disturbs the proximal growth plate (GP) of tibia bone in chickens. Normal GP development entails cartilage vascularization and mineralization followed by osteogenesis. However, TD is characterized by tibial metaphyseal cartilage cell proliferation, non-vascular, non-mineralized, and white opaque mass in the GP (Li et al., 2008; Shahzad et al., 2014; Nabi et al., 2016a). TD affected chickens show depression, reduction in physical activities, feeding and drinking, standing difficulty, gait inflexible, movement disorders, and wings support walking (Jefferies et al., 2000; Shahzad et al., 2014). Under normal conditions, TD occurred during the development of GP, and most of the cases appeared with unobvious symptoms at early stages. However, at later stages the tibia bone become fragile; cause tarsi bending, deformity, or tibial fractures (Rath et al., 2007; Tian et al., 2013). TD is the most common and abnormal bone disease, which reduces the yield and quality of meat in chickens, ultimately causes economic losses to poultry industry (Edwards, 2000; Li et al., 2008; Dan et al., 2009).

Normal chicken bone development starts from the immature cartilage cells of static zone, then proliferative zone, hypertrophy stage and gradually mature, angiogenesis, calcification, and finally bone tissue formation (Rath et al., 2007; Borjesson et al., 2013). However, the excessive growth was found in immature cartilage with cells arrange tightly, osteoclasts and osteoblasts scarce in proliferative zone. Some nucleus psychosis and apoptosis was seen in severe tibial GP lesions (Pines and Hurwitz, 1991; Velada et al., 2011; Shahzad et al., 2014). Due to a lack of blood vessels that cannot provide the corresponding nutrients, lead to no calcification in cartilage and disordered arrangement in hypertrophic zone and metaphasis (Pines et al., 1998, 2005; Kronenberg, 2003).

The etiology of TD is associated with varieties of factors such as growth rate, environment, sex, toxins, and composition of the diet. Furthermore, induction of TD lesion for instance by addition of thiram, tumor necrosis factor, interleukin-1, the growth factor, and the fungicide causing TD due to interruption in GP metabolism and development of chondrocytes (Shim et al., 2012; Tian et al., 2013). However, further molecular studies show that the occurrence of TD is associated with vascular endothelial growth factor (VEGF), bone loss, bone resorption, and osteoclast formation (Kronenberg, 2003; Rath et al., 2007). Therefore, our study adopts the methods of molecular biology affecting VEGF and wingless-type member 4 (WNT4) genes associated with bone calcium absorption. The WNT4 is key regulator in early embryogenesis; stem cells and WNT signaling are involved in the regulation of numerous processes of cell proliferation, differentiation and morphogenesis in humans and other species (Timmermans-Sprang et al., 2017). The WNT4 is a secreted protein from osteoblasts and it has significant affect to inhibit bone resorption (Hartmann and Tabin, 2000; Algandaby et al., 2017). The VEGF and its receptors are pro-angiogenic regulators; VEGF is essential mediator during bone development processes, blood vessel growth and GP chondrocytes differentiation (Zhang et al., 2013). The cellular morphology of different regions of GP revealed that TD is characterized by significantly thickened avascular cartilage plug, dry chondrocytes, empty cartilage lacunae, and lack of blood vessels in the GP (Gerber and Ferrara, 2000; Kronenberg, 2003; Dan et al., 2009; Genin et al., 2012; Zhang et al., 2013).

**FIGURE 1:**
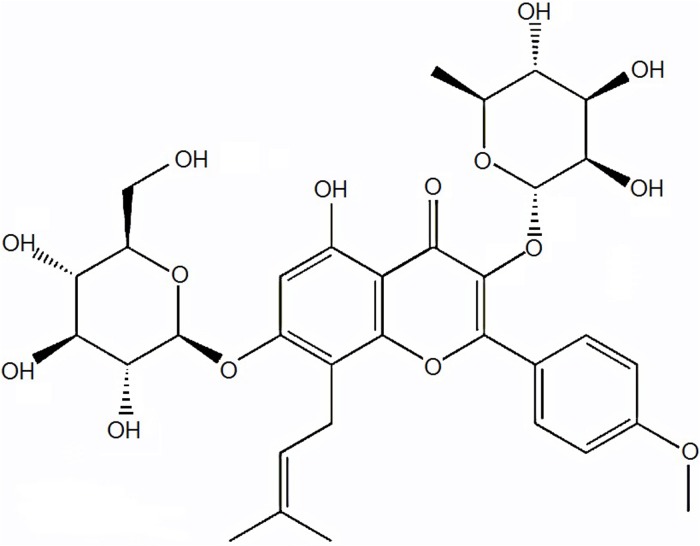
Chemical structure of icariin compound.

Icariin (C_33_H_40_O_15_; molecular weight: 676.65; **Figure 1**) is a natural flavonoid glycoside isolated from some species of plants belonging to the genus *Epimedium* (Schluesener and Schluesener, 2014). Various researchers indicated that icariin has antioxidant, anti-inflammatory, anti-angiogenic, and anti-autophagic properties (Li et al., 2015; Ye et al., 2015; Mo et al., 2016; Algandaby et al., 2017). It is suggested to be a potential accelerator for cartilage tissue engineering (Li et al., 2012; Zhang et al., 2012). Recently, icariin got much attention due to the potential to treat bone diseases as well as inhibit bone resorption. Considering the characteristics of icariin, we hypothesized that it can prevent TD by improving bone formation and oxidative imbalance. Therefore, the purpose of the present study was to investigate the effect of icariin to protect thiram-induced TD in broiler chickens via regulations of WNT4 and VEGF expressions.

## Materials and Methods

### Animal Ethics and Welfare

All the experiments were conducted according to protocols approved by Institution’s Animal Care and Health and Supervisory Committee of Huazhong Agricultural University, Wuhan, China (approval number: 31460682).

### Chicken Management and Experimental Design

One hundred and eighty 1-day-old Arbor Acres broiler chickens (weighing 48 ± 6 g) were selected from a commercial hatchery. The chickens were equally divided into three groups (*n* = 60); control, TD, and icariin. All groups were offered *ad libitum* regular diet as suggested by the National Research Council (2001) as mentioned in Supplementary Table S1. The TD and icariin group were fed same diet as control group but with the addition of tetramethylthiuram disulfide (thiram) 50 mg/kg of feed from days 3 to 7 posthatch to induce TD. After induction of TD on day 8, the icariin group was separated and fed with standard normal diet with the addition of 10 mg/kg/day icariin (Xiao et al., 2015) through drinking water until the end of experiment, while TD group was given standard normal diet without adding thiram, just like control group.

### Sample Collection and Production Parameters Analysis

All groups were reared for 18 days and number of lame birds, daily weight gain, feed intake, and feed conversion ratio (FCR) in every group was calculated on various days. During the experiment period, 15 chickens from each group were randomly selected to sacrifice them by cervical dislocation on days 7, 10, 14, and 18. Chicken blood was collected for biochemical analysis just before the slaughtering. After that, tibia bone parameters including tibia weight was calculated by an electronic balance, while tibia length, width, and width of tibial GP was measured by digital calipers (SATA91511, TATA Company, Shanghai, China). Then some of the tibia bones were fixed in paraformaldehyde (4%) for hematoxylin and eosin (H&E) staining and others were frozen in liquid nitrogen and stored at -70°C for further analysis through reverse transcription and quantitative real-time polymerase chain reaction (RT-qPCR) and western blotting. The liver samples were also dissected out, and stored at -70°C. For the morphological examination, the tibia bones in each group were dissected with scalpel blade and morphological examination of GP was performed.

### Serum Biochemical Analysis

The blood samples were centrifuged at 3000 × *g* for 20 min to collect the blood serum and stored at -20°C. The concentration of aspartate aminotransferase (AST), alkaline phosphatase (ALP), and alanine aminotransferase (ALT) in the serum samples were measured in control, TD, and icariin groups by using commercial reagent kit (Nanjing Institute of Biological Engineering Inc., Jiangsu, China) via semi-automatic biochemical machine (COULTER^®^LH 750, Guangdong). The activities of serum biochemical parameters were presented in unit per liter (U/L).

### Measurement of Liver Antioxidant Enzymes

The liver samples were processed for measuring the glutathione peroxidase (GSH-Px), superoxide dismutase (SOD), total antioxidant capacity (T-AOC), and malondialdehyde (MDA) contents by a commercial assay kit (Nanjing Institute of Biological Engineering Inc., Jiangsu, China) in control, TD, and icariin groups according to Nabi et al. (2016a). Briefly, the livers in each group were rinsed and measured, then homogenized for 10 min and samples were centrifuged at 3000 × *g* for 10 min at 4°C to assess the contents.

### Hematoxylin and Eosin (H&E) Staining

The tibia bones (*n* = 10) from each group were stored in formalin solution and processed for H&E staining according to our previous study (Mehmood et al., 2018).

### RNA Extraction and RT-qPCR

Total RNA of GPs (*n* = 10) from each group was collected using Trizol reagent (Invitrogen, Carlsbad, CA, United States), and we translated the RNA into cDNA by cDNA kit (Tian Gen, China) as per manufacturer’s guideline. The WNT4 and VEGF primers were designed by Primer Premier Software (version 5.0) and ordered to Wuhan Qingke Biotechnology Co., Ltd., China (**Table 1**). The qRT-PCR was run in quadruplex with Step One-Plus^TM^ Real-Time PCR System (Applied Biosystems). The reaction mixture specifications were according to our previous study (Chang et al., 2017). All the reaction mixture was normalized against the reference gene GAPDH.

**Table 1:** Primers used for the quantitative polymerase chain reaction.

Genes	Accession number	Primer sequence (5′–3′)	Product size (bp)	Tm (°C)
*VEGF*	XM_019612783	F: AAAGCGAGGAAAGGGGAAGG	94	55
		R: TCTCCTCTCTGAGCAAGGCT		
*WNT4*	NM_204783.1	F: TGTGACCACGACCTCAAGAA	160	59
		R: ACCAGTGGAATTTGCAGCTG		
*GAPDH*	XM_019960295	F: CCTTCATTGACCTTCACTACATGGTCTA	127	58
		R: TGGAAGATGGTGATGGCCTTTCCATTG		


### Western Blot Analysis

First of all GP tissues (*n* = 10) from each group were homogenized in PBS (ice-cold) and kept for 2 h at 4°C. After that, centrifuged the material at 14,000 rpm for 10 min and supernatant was collected. The concentration of the total proteins was calculated by Coomassie Brilliant Blue g-250 method. The equal amounts of protein were subjected to 10% SDS polyacrylamide gel and transferred onto PVDF membrane. The membrane was incubated in 5% skim milk at room temperature for 1.5 h and subsequently treated with primary antibody (1:1000 dilution) at 4°C for overnight. Then membranes were washed three times with Tris-buffered saline containing 0.1% Tween 20 (TBST) for 5 min and incubated with goat anti-mouse secondary antibody (1:5000 dilution) at room temperature for 30 min (Tuojie Biological Technology Co., Ltd., Wuhan). The membranes were again washed four times with TBST. The image was taken with an imaging system (UVP, Upland, CA, United States).

### Statistical Analysis

The results were evaluated through two-way ANOVA and Student’s *t*-test by using SPSS 19.0 software. The differences were considered statistically significant if *P* < 0.05.

## Results

### Morphological and Clinical Observation of Thiram-Induced TD

**FIGURE 2:**
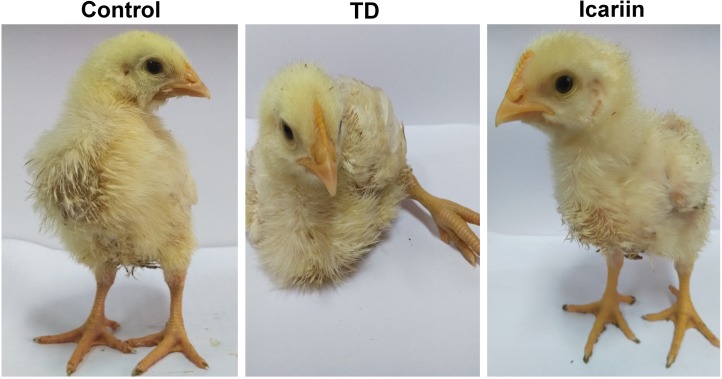
Effects of icariin on thiram-induced tibial dyschondroplasia-dependent lameness.

The visual assessment indicated that chickens were showing depression with poor body condition in weakness, lameness, and feeding difficulties in TD group. Compared with the TD affected chickens, the icariin treatment group regained their ability to stand and walk properly (**Figure 2**). The continuous infusion of icariin caused a further decrease in signs of lameness, and almost all birds reverted to the control levels especially on day 18.

### Effect of Icariin on Performance of Chickens

**FIGURE 3:**
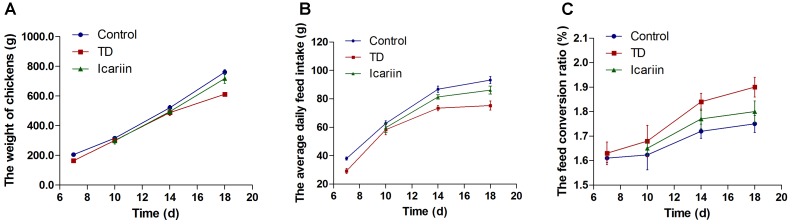
Overall performance parameters analysis of chicken among three groups. **(A–C)** Correlation analysis among weight of chickens, average daily feed intake, and feed conversion ratio (FCR) were recorded with Pearson test. Overall, the body weight and average daily feed intake in icariin group were increased as compared with TD group, especially after day 10. At the same time, the FCR had a lower value as compared with TD group. Icariin group approach to control group compared with TD group. The standard value of FCR is 1.6 in this study.

Overall performance parameters of chickens are presented in **Figure 3**. The results indicated that there was a positive correlation between weight of chickens and average daily feed intake, while negative association in FCR in TD and icariin groups. The daily weight gain and average daily feed intake were increased while FCR were decreased in icariin groups compared with TD groups. The FCR was above the standard value (1.60) in TD afflicted group as compared to control group. Whereas, administration of icariin results in reduction in FCR near to standard value as compared to TD group. Altogether, icariin-treated chickens were looking healthy, active, and alert than TD group. Additionally, recovery in icariin-treated chickens group was much higher than TD group chickens.

### Effect of Icariin on Tibial Bone Morphometry

**FIGURE 4:**
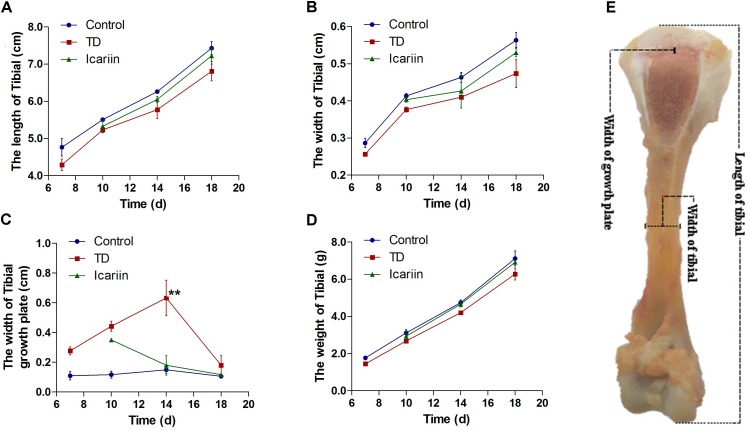
The overall parameters analysis of tibia bone. **(A–D)** Correlation analysis among length of tibia, width of tibia, weight of tibia, and width of tibial growth plate were recorded with Pearson test during the experiment from days 7 to 18. **(E)** Measurement of width of growth plate, width of tibia and length of tibia bone. ^∗∗^*P* < 0.01.

**FIGURE 5:**
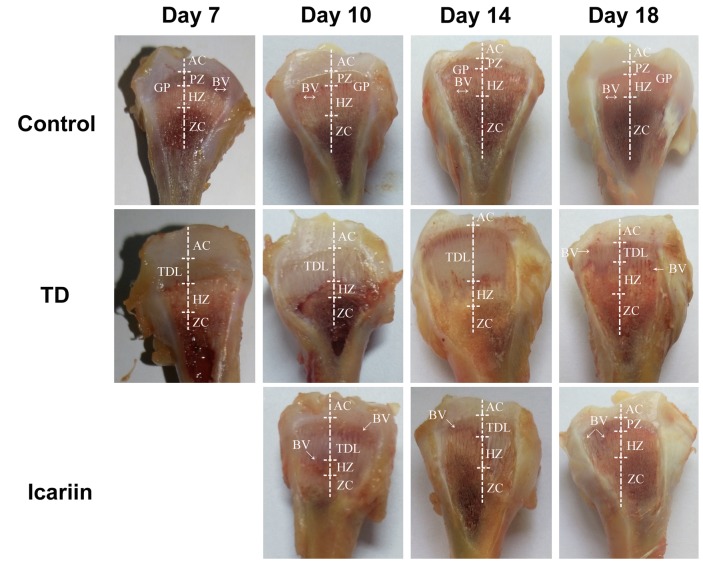
The Morphological analysis of growth plates of control, TD, and icariin-treated chickens during the experiment from days 7 to 18. Extended GP was compared in TD group with control and icariin group. AC, articular cartilage; BV, blood vessel; GP, growth plate; PZ, proliferative zone; HZ, hypertrophic zone; TDL, tibial dyschondroplasia lesion; ZC, zone of calcification.

Tibial bone performance indicators are presented in **Figure 4**. The results indicated that there was positive correlation among length, width, and weight of tibia bone while negative association in width of GP in TD and icariin groups. The length, width, and weight of tibia bone were decreased in TD group but the difference was not significantly (*P* > 0.05), while GP width and TD score were increased significantly (*P* < 0.05) in TD affected thiram group from days 7 to 14 as compared to control. However, icariin administration resulted in a significant reduction (*P* < 0.05) of TD score and GP width as compared to thiram-fed TD group chickens (**Figure 5**). Furthermore, TD lesions diminished and blood vessels developed in tibial GP, while chickens regained their ability to stand and walk properly in icariin-treated group.

### Histological Examination of Tibial Growth Plates

**FIGURE 6:**
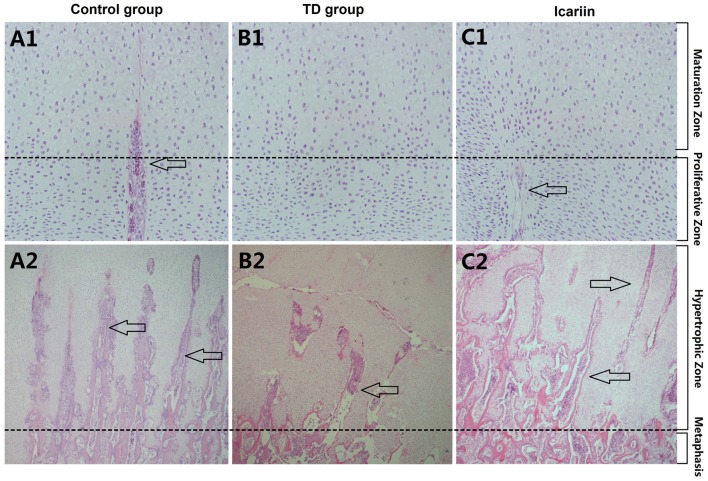
H&E stained histopathology of epiphyseal tibial growth plate (GP). **(A1,A2)** The normal GP exhibits regular columns of cells surrounded by blood vessels in proliferative and hypertrophic zone. **(B1,B2)** Necrosis and reduced number of blood vessels and avascularized GP in thiram-fed birds in TD group. **(C1,C2)** Restored angiogenesis in proliferative and hypertrophic zones of growth plate shown in icariin group. Arrows indicate blood vessels.

Histology of normal tibial GPs revealed well-conserved columns of cells surrounded by large numbers of blood vessels in proliferative and hypertrophic zones of tibial GP (**Figure 6**). However, in thiram-fed TD group chickens, chondrocytes were narrowed and columns of cells became asymmetrical and necrotized, while less number of blood vessels was seen in tibial GP as compared to control group. Whereas, icariin treatment restored the width of the hypertrophic region, angiogenesis was observed, and GP appeared normal as compared to TD group (**Figure 6**).

### Effect of Icariin on Serum Biochemistry

**FIGURE 7:**
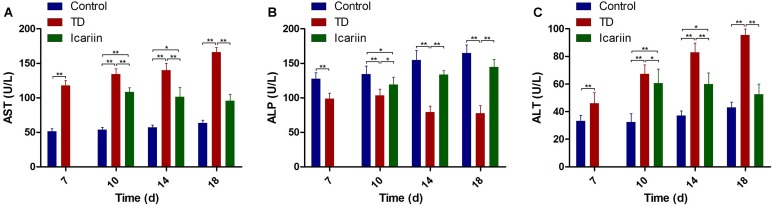
Serum-biochemical analysis of control, TD, and icariin group on days 7, 10, 14, and 18. **(A–C)** Serum-biochemical analysis of AST, ALP, and ALT in control, TD and icariin groups on various days. The data are presented in mean ± SD. ^∗^*P* < 0.05, ^∗∗^*P* < 0.01.

Our experiment showed significant decrease (*P* < 0.05) in ALP activity along with significant increased (*P* < 0.05) in ALT and AST levels in blood serum of TD group throughout the experiment period on days 7, 10, 14, and 18, as compared to control. However, icariin therapy restored ALP, ALT, and AST levels and normalized these contents to control group and there was no significant difference (*P* > 0.05) between icariin and control groups on day 18. Conversely, there was significant difference (*P* < 0.01) in ALP, ALT, and AST levels between icariin and TD group on days 7, 10, and 18 (**Figure 7**).

### Effect of Icariin on Liver Antioxidant Enzymes

**FIGURE 8:**
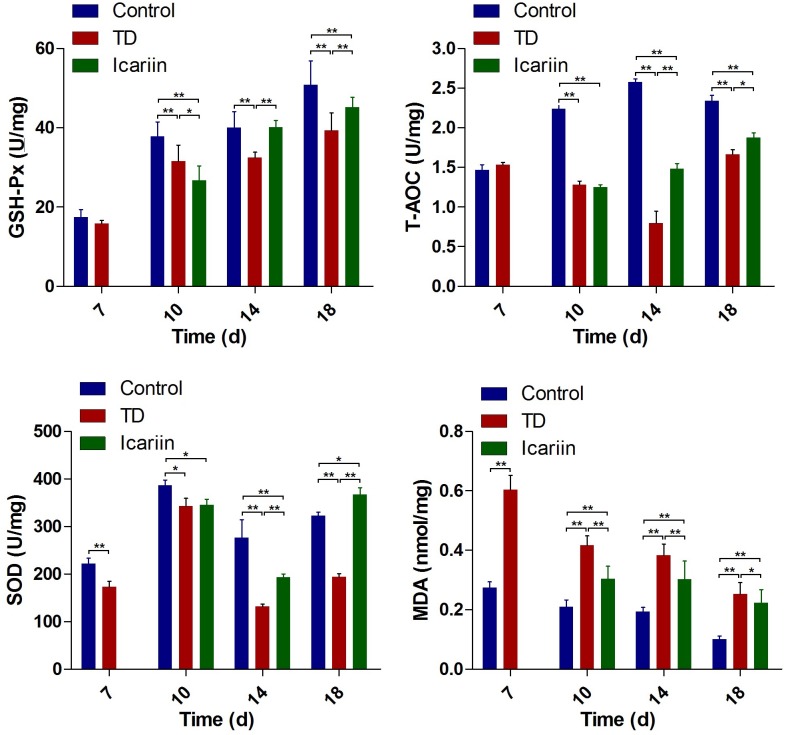
The antioxidant activities analysis of liver in control, TD, and icariin group chickens at 7, 10, 14, and 18 days. The data are presented in mean ± SD. ^∗^*P* < 0.05, ^∗∗^*P* < 0.01.

The antioxidant levels in liver indicated that antioxidant enzymes SOD, T-AOC, and GSH-Px decreased significantly (*P* < 0.05), while MDA contents were increased in thiram-induced TD group as compared to control. However, icariin therapy diminished oxidative imbalance by increasing SOD, T-AOC, and GSH-Px level and decreasing MDA contents significantly (*P* < 0.05) in icariin group as compared to TD group (**Figure 8**).

### The WNT4 and VEGF Genes Expressions in Growth Plate

**FIGURE 9:**
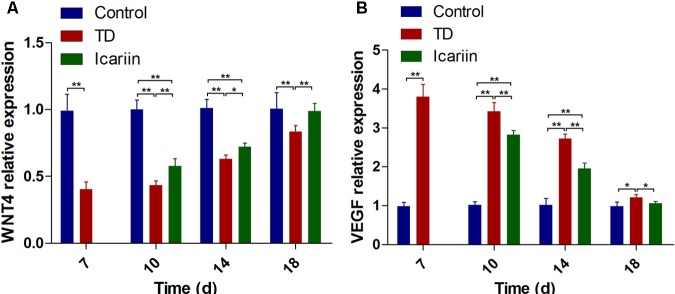
RT-qPCR analysis of WNT4 and VEGF genes in growth plate cells was evaluated in control, TD, and icariin group chickens at 7, 10, 14, and 18 days. **(A)** The mRNA levels of WNT4 was detected by RT-qPCR. **(B)** VEGF mRNA levels detected by RT-qPCR. Correlation coefficient = target gene (average optical density)/reference gene (average optical density). The results are presented in arbitrary units as means ± SEM. ^∗^*P* < 0.05, ^∗∗^*P* < 0.01.

The expression of WNT4 and VEGF genes in tibial GP of chickens was confirmed by RT-qPCR. Our results indicated that mRNA expression of WNT4 gene was significantly decreased (*P* < 0.01) in TD group as compared to control group on days 7, 10, 14, and 18. Whereas, administration of icariin significantly up-regulated the WNT4 gene expression (*P* < 0.05) on days 10–18 compared to TD group chickens. Furthermore, expression of WNT4 gene in icariin treatment group was at the level of control group on day 18 and difference was not significant between icariin and control group chickens (**Figure 9A**). The VEGF expression depicted a significant up-regulation (*P* < 0.01) from days 7 to 18 in TD affected birds as compared to control group. However, administration of icariin significantly decreased (*P* < 0.01) VEGF expression from days 10 to 18 posthatch compared to TD group (**Figure 9B**). Moreover, expression of VEGF gene in icariin treatment group was near the level of control group on day 18.

### Effect of Icariin on WNT4 and VEGF Proteins Level

**FIGURE 10:**
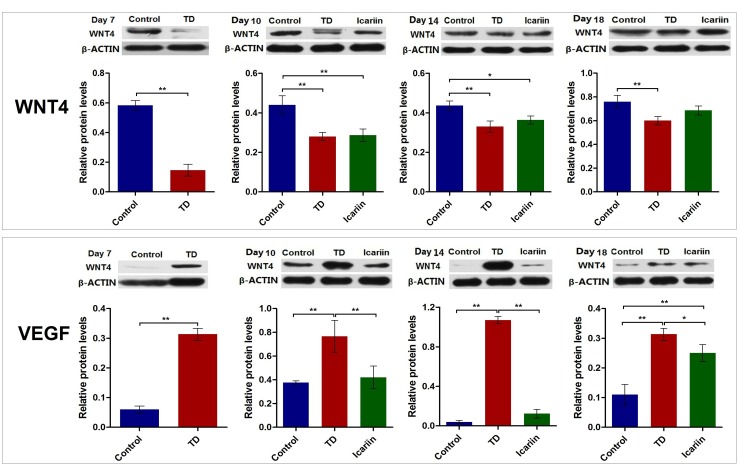
The WNT4 and VEGF protein level was analyzed in tibial growth in control, TD, and icariin group chickens at 7, 10, 14, and 18 days. Data is expressed in arbitrary units as means ± SEM. ^∗^*P* < 0.05, ^∗∗^*P* < 0.01.

The WNT4 and VEGF protein levels were assessed by western blotting analysis in tibia bone on days 7, 10, 14, and 18. Results revealed that WNT4 expression was significant down-regulated (*P* < 0.05) in TD group on days 7, 10, 14, and 18 compared with control group. Whereas, icariin administration obviously increased the expression levels of WNT4 protein on days 14 and 18 compared to TD group, and there was no prominent difference in the expression of WNT4 protein between icariin and control group on day 18 (**Figure 10**). The VEGF protein level was significant up-regulated (*P* < 0.05) in TD chicken throughout the study period as compared to control group. However, icariin therapy decreased the protein level of VEGF significantly (*P* < 0.05) from days 10 to 18 (**Figure 10**) compared to TD group.

## Discussion

Tibial dyschondroplasia is mainly a cartilage metabolic disease with leg problem in fast growing birds that affect the proximal tibial GP. TD is described as non-mineralized and avascularized cartilage with extended GP, tibia bone deformation and lameness in chickens (Jefferies et al., 2000; Rath et al., 2007). Previous reports indicated that the main clinical features of TD are gait abnormality; less feed intake, single or both legs lameness, ataxia, inability to stand, and ultimately death (Tian et al., 2013; Shahzad et al., 2014; Nabi et al., 2016a). TD has very big influence on yield and quality of meat in chicken; it causes serious economic losses to the poultry industry (Shahzad et al., 2014; Nabi et al., 2016a). Icariin is a proficient compound for the anti-osteoporosis and restoration of bone defects (Chen et al., 2005; Zhao et al., 2010). Icariin may effect on bone regeneration by enhancing the endochondral ossification (Zhao et al., 2010). Experimentally, it has been proved that icariin possess antidepressant properties (Wei et al., 2016).

Our current observations confirmed the previous reports that TD reduced body weight gain. Overall, daily weight gain and average daily feed intake in icariin treatment group were clearly increased as compared with TD group, especially after day 14. At the same time, the FCR was superior as compared with TD group. TD group chicks faced difficulties in walking, uncomfortable body movement, incapable to stand, less feed intake, less weight gain, and poor FCR, which seriously affect the production performance of broilers. Our findings indicated that the ability of walking, sports ability, and growth was gradually recovered in icariin-treated chickens as compared with TD group from days 10 to 18. Additionally, the recovery rate and degree of growth performance was improved after icariin treatment. Altogether, icariin therapy showed significant influence in the treatment of TD.

GP cartilage has a specific morphology and distinct zonal arrangements. Each region of the GP is populated with mature cells or cartilage cells (Rath et al., 2007; Velada et al., 2011; Shim et al., 2012). Chondrocytes are specialized active cells, and they are arranged in columns to form the distinct zones parallel to long axis of the bone with well-structured morphology (Domowicz et al., 2009). Chondrocytes have very stable morphology and TD results in some abnormal modification in the mechanism and action of chondrocytes, which causes various histological changes. In our study, we observed that GP have regular columns of cells bounded by huge number of blood vessels in hypertrophic and proliferative zones in control group. Whereas, TD afflicted thiram-fed chickens tibial GP showed less number of blood vessels, necrotized, narrow and asymmetrical columns of cells with non-vascularized and immature cartilage chondrocyte, while vascular osteoclasts and osteoblasts were less in proliferative zone along with some nucleus pycnosis and apoptosis. However, restored angiogenesis in proliferative and hypertrophic zones of GP was considerable after the administration of icariin for the treatment of TD. Wang et al. (2016) studied that icariin promoted the cartilage tissue repair by regulating the chondrocytes propagation and differentiation with bone formation and mediated bone-protective effects. Icariin is a proficient growth factor that reduces the hypertrophic differentiation and increases the chondrocytes differentiation in cartilage tissue (Wang et al., 2014).

The AST and ALT are indicators to evaluate the liver functions and more AST and ALT values indicate that aminotransferase release from liver cells into the blood due to liver damage. Icariin significantly reduced ALT and AST leakage from rat liver cells (Algandaby et al., 2017). Thiram is considered an effective oxidative agent, and it inhibits SOD and GSH activity in human which lead to oxidative stress (Marikovsky, 2002). The oxidative stress leads to release of MDA and lipid peroxidation of cell membrane (Perry et al., 2010; Shahzad et al., 2014). Our investigation regarding the changes of antioxidant indicators in the liver of TD and icariin treatment chickens indicated that SOD, T-AOC, and GSH-Px values were remarkably decreased in TD group, while MDA contents were considerably increased in TD group. Icariin treatment restored antioxidant capability by considerable increasing in SOD, T-AOC, and GSH-Px values and decreasing the level of MDA. Algandaby et al. (2017) also reported same result that after the treatment of icariin in thioacetamide-induced liver fibrosis in rats, SOD, MDA, and GSH-Px contents were restored to normal values in rats. Our results indicated that icariin exhibited antioxidant properties, because it corrected the damaging effects on liver by restoring the oxidative imbalance in broiler chickens. Experimentally, it has also been reported previously that icariin possess antioxidant activities (Ke et al., 2015).

Previous reports showed that transgenic mice over expressing WNT4 from osteoblasts demonstrated a protective effect of WNT4 on bone loss and chronic inflammation by inhibiting osteoclastogenesis and bone resorption (Yu et al., 2014). Hartmann and Tabin (2000) showed that the WNT4 from osteoblasts significantly protected bone loss, inhibited osteoclast formation and bone resorption. In our study, WNT4 level was significantly decreased in TD afflicted chickens on days 7 to 14. The WNT4 was discovered to play an important role in proliferation and migration in vascular smooth muscle cells (Li et al., 2017), which revealed that TD induced by thiram was related to down-regulation of WNT4 through decrease in proliferation and migration of vascularization. The WNT signaling pathway plays main role in osteoblast differentiation and bone formation, but if there is any abnormal level during the pathway then it causes development of pathological conditions in bone (Lee et al., 2012; Wang et al., 2016). Our results demonstrated that expressions level of WNT4 was restored by icariin on day 18 as compared with TD chickens. Icariin has bone-protective action by preventing bone loss, stimulated bone formation and increased bone mass (Zhang et al., 2007; Nian et al., 2009). Icariin significantly increased the expression level of Wnt1, and Wnt3a in mice (Li et al., 2013). The growth and development of the bone cannot be achieved and sustained unless from the nutritional support of blood (Filipowska et al., 2017). The VEGF is the critical pro-angiogenic factor for vascular formation (Zhang et al., 2013; Le et al., 2015). Thiram appeared to cause up-regulation of VEGF expression, suggesting that it has proangiogenic activity (Rath et al., 2005). Our study found a significant increased in mRNA and protein expression level of VEGF on days 7 to 14 in TD chickens. Our findings of up-regulation of VEGF expression are concomitant with previous reports (Herzog et al., 2011; Shahzad et al., 2015; Nabi et al., 2016b; Mehmood et al., 2017). Dan et al. (2009) and Genin et al. (2012) described that TD endorsed the change in VEGF signaling pathways and abnormal chondrocyte differentiation. Icariin therapy down-regulated the expression of VEGF in TD chickens which is consistent with previously reported inhibition of VEGF in mice by icariin (Algandaby et al., 2017). Many researchers report that icariin is antioxidant, anti-angiogenic, and anti-inflammatory effects (Li et al., 2015; Ye et al., 2015; Mo et al., 2016). The antiangiogenic activity of icariin was substantiated by assessing VEGF gene and protein expression. Algandaby et al. (2017) also reported similar results that icariin significantly inhibited the up-regulation of VEGF expression in rats.

Currently, many drugs have been used for the treatment of bone-related abnormalities in chickens and this study clearly suggested that icariin prevent avascularized GP by inducing new blood vessels formation and promotes normal differentiation and mineralization of proximal avian GP chondrocytes by up-regulating the expressions WNT4. Icariin regulates WNT4 and VEGF expressions for proper chondrocyte differentiation, diminish lameness and attenuate TD in broiler chickens. In conclusion, icariin restores the GP width, increase growth performance, corrected liver functions and antioxidant enzymes levels in liver, and mitigated the lameness in broiler chicken. Therefore, icariin therapy is encouraged based on evidence rationale for the treatment of TD in broiler chickens.

## Author Contributions

HZ and KM made equal contributions in conducting experiments. JL, HZ, and KM provided the research idea. MR, KL, FN, XJ, SH, LW, LZ, XT, WY, MI, and MS contributed reagents, materials, and analysis tools. HZ and KM wrote the manuscript. All the authors participated in writing and reviewing the manuscript.

## Conflict of Interest Statement

The authors declare that the research was conducted in the absence of any commercial or financial relationships that could be construed as a potential conflict of interest.
