# Supplementary material for: Icariin Ameliorate Thiram-Induced Tibial Dyschondroplasia via Regulation of WNT4 and VEGF Expression in Broiler Chickens
Source: Front Pharmacol. 2018 Feb 23;9:123. doi: 10.3389/fphar.2018.00123 (PMC5829035; doi:10.3389/fphar.2018.00123)
Supplement: Supplementary file 1 [file Table_1.DOC]

**Table S1:** Composition and nutrient levels of the basal diet

| Ingredients | Contents (%) | Nutrient levels | Content |
| --- | --- | --- | --- |
| Corn | 63.8 | Digestive energy, MJ/kg | 12.02 |
| Soybean | 28.0 | Crude protein, % | 19.80 |
| Fish powder | 2.5 | Calcium, % | 0.90 |
| CaHPO_4_ | 1.4 | Phosphate, % | 0.47 |
| NaCl | 0.3 | Lysine, % | 1.02 |
| premix | 4.0 | Methionine, % | 0.34 |

Note: The premix provided the following per kg of diets: Mn 66mg, Zn 44mg, Cu 9mg, Fe 50mg, I 0.4mg, V_A_ 7000 IU, V_D3_ 875 IU, V_E_ 20 IU, V_K3_ 1mg, V_B1_ 2mg, V_B2_ 4.5mg, V_B6_ 2.5mg, V_B12_ 0.6mg.
